# Health care quality measures for children and adolescents in Foster Care: feasibility testing in electronic records

**DOI:** 10.1186/s12887-018-1064-4

**Published:** 2018-02-22

**Authors:** Katherine J. Deans, Peter C. Minneci, Kristine M. Nacion, Karen Leonhart, Jennifer N. Cooper, Sarah Hudson Scholle, Kelly J. Kelleher

**Affiliations:** 1 The Research Institute at Nationwide Children’s Hospital, 700 Children’s Drive, FB3145, Columbus, OH 43205 USA; 2 National Quality Forum, 1030 15th Street NW, Suite 800, Washington, DC 20005 USA; 3 National Committee for Quality Assurance, 1100 13th St, NW, Suite 1000, Washington, DC 20005 USA

**Keywords:** Foster care, Quality measures, Electronic health record, Statewide automated child welfare information system

## Abstract

**Background:**

Preventive quality measures for the foster care population are largely untested.

The objective of the study is to identify healthcare quality measures for young children and adolescents in foster care and to test whether the data required to calculate these measures can be feasibly extracted and interpreted within an electronic health records or within the Statewide Automated Child Welfare Information System.

**Methods:**

The AAP *Recommendations for Preventive Pediatric Health Care* served as the guideline for determining quality measures. Quality measures related to well child visits, developmental screenings, immunizations, trauma-related care, BMI measurements, sexually transmitted infections and depression were defined. Retrospective chart reviews were performed on a cohort of children in foster care from a single large pediatric institution and related county. Data available in the Ohio Statewide Automated Child Welfare Information System was compared to the same population studied in the electronic health record review. Quality measures were calculated as observed (received) to expected (recommended) ratios (O/E ratios) to describe the actual quantity of recommended health care that was received by individual children.

**Results:**

Electronic health records and the Statewide Automated Child Welfare Information System data frequently lacked important information on foster care youth essential for calculating the measures. Although electronic health records were rich in encounter specific clinical data, they often lacked custodial information such as the dates of entry into and exit from foster care. In contrast, Statewide Automated Child Welfare Information System included robust data on custodial arrangements, but lacked detailed medical information. Despite these limitations, several quality measures were devised that attempted to accommodate these limitations.

**Conclusions:**

In this feasibility testing, neither the electronic health records at a single institution nor the county level Statewide Automated Child Welfare Information System was able to independently serve as a reliable source of data for health care quality measures for foster care youth. However, the ability to leverage both sources by matching them at an individual level may provide the complement of data necessary to assess the quality of healthcare.

## Background

Demands for information on the quality of pediatric preventive care have spurred investment in the development of quality measures designed to access the current state of children’s health care and ultimately define areas for improvement [1]. Specific quality measures for the foster care population, though, are largely untested [2, 3], even though youth entering foster care have greater physical, developmental, and mental health needs than their peers in the general population [4, 5]. Although children in foster care are known to have higher rates of social and medical morbidity, guidelines for the care of foster children are rarely adhered to in routine practice and may be difficult to measure [6–10]. One of the most important ways to improve care and reduce poor long term health outcomes is through the development and testing of reliable quality measures for these high risk youth. To date, measurement has largely been dependent on labor-intensive chart reviews. Two other possible sources of quality data that might be extracted electronically exist for foster care children [11]. A clinical electronic health record (EHR) has potential to provide comprehensive and detailed patient-level data with electronic extraction on a large population. In addition, the Statewide Automated Child Welfare Information System (SACWIS) is a database used by protective services agencies to hold the official case records of children in care. Many states hold health data in their SACWIS records.

In order for these datasets to be useful, each of them would have to contain sufficient detail on health services to compare them against recommended guidelines, entry and exit dates for foster care to calculate eligibility, and demographic data for stratification. The primary objectives of this study were to identify quality measures, such as the appropriate number of well care visits, vaccinations and developmental screening for young children (ages 0–3) and adolescents (ages 12–18) in foster care and test whether the data required to calculate these measures can be feasibly interpreted within an EHR or within SACWIS. These two age groups were chosen because of the diversity of well care measures available for testing in both groups.

## Methods

This work was performed as part of the Children’s Health Insurance Program Reauthorization Act (CHIPRA) Pediatric Quality Measures Program (PQMP), specifically as part of the National Collaborative for Innovation in Quality Measurement (NCINQ). Our approach considered three time periods of child welfare engagement: (1) entry into foster care, (2) ongoing foster care, and (3) foster home change or exit. [12–24] We gathered input from a national advisory panel representing foster care alumni, national policy makers, state child welfare and Medicaid officials, health plan staff, and academic researchers.

Retrospective chart reviews were performed at Nationwide Children’s Hospital (NCH). For the study of children aged 0 to 3 years, we abstracted data from the time period of January 1, 2007- February 28, 2013 from children who met inclusion criteria: 1) In foster care (not including kinship care) within Franklin County, Ohio, and 2) at least one comprehensive well-care visit at a primary care physician (PCP) clinic or foster care specialty clinic at NCH. For the study of children aged 12–18 years, we abstracted data from the time period of January 1, 2009- October 31, 2013 with the same criteria. All extracted data was for care that occurred while the child was in foster care. We defined foster care as full-time care provided by an approved foster care family or group home and excluded any care provided by kin or close family friends (Table 1).

**Table 1:** Calculation of Proposed Quality Measures for Children in Foster Care within an EHR: Proportion Measures

Measure	Numerator	Denominator	Guideline(s) Referenced	Notes
Young children (age 0–3 years)				
1st well-care visit within 30 days of initial entry into foster care	Children with a first well-care visit within 30 days of entry	All children with an exact date or date range of entry into foster care	Health Care of Young Children in Foster Care [23]	Only children with an exact date or date range of initial entry into foster care are included in the denominator
Appropriate number of well care visits for their age during foster care	Children who received all recommended well-care visits for their age during foster care	All children	Recommendations for Preventive Pediatric Health Care [24]	Well-care visits were not required to occur within any particular window of time around the exact ages of recommended well-care visits
Appropriate number of vaccinations by age 1	Children with all recommended vaccinations received by 1 year of age, whether received while in foster care or not	All children who turned 1 year of age while in foster care	CDC 2013 Immunization Schedules [32] HEDIS Childhood	HEDIS requirements for the appropriate timing of vaccines as specified for their Combination #2 measure were followed. Only those vaccinations that are supposed to occur by age 1 were included (i.e. DTaP, IPV, HiB and HepB)).
			Immunization Status [29]	
Appropriate number of vaccinations by age 2	Children with all recommended vaccinations received by 2 years of age, whether received while in foster care or not	All children who turned 2 years of age while in foster care	CDC 2013 Immunization Schedules [32] HEDIS Childhood	HEDIS requirements for the appropriate timing of vaccines as specified for their Combination #2 measure were followed. Only those vaccinations that are supposed to occur by age 2 were included (i.e. DTaP, IPV, HiB, HepB, MMR and VZV)
			Immunization Status [29]	
Appropriate number of lead screenings for their age during foster care	Children who received the appropriate number of lead screenings while in foster care	All children who turned 1 year and/or 2 years of age while in foster care	Guidelines for Medicaid Lead Testing [33]	Screenings had to occur during the following age periods, if the child was in foster care at age 1 year and 2 years respectively: 9–21 months of age and 22–36 months of age
Appropriate number of developmental screenings for their age during foster care	Children who received all recommended developmental screenings for their age during foster care	All children	Recommendations for Preventive Pediatric Health Care [24]	Screenings were not required to occur within any particular window of time around the exact ages of recommended screenings. Documentation that a specific standardized tool used was not necessary; any mention of a developmental screening was included.
Developmental screening during foster care within 3 months after documentation of traumatic brain injury (TBI)	Children who received a developmental screening within 3 months after documentation of TBI	All children diagnosed with a TBI prior to entry into foster care	Evaluation of suspected child physical abuse [21]	A 3 month window was used based on professional medical opinion
			Head Injury. Triage, Assessment, Investigation and Early Management of Head Injury in Infants, Children and Adults [13]	
Follow up skeletal survey after receiving an initial skeletal survey	Children who received a follow up skeletal survey	All children who received an initial skeletal survey	Evaluation of suspected child physical abuse [21]	The follow up skeletal survey was not required to occur within any certain period of time after the initial skeletal survey
Care coordination letters at foster home changes	Instances of foster home changes that had evidence of a care coordination letter	All documented foster home changes	Health Care of Young Children in Foster Care [23]	
Adolescents (age 12–18 years)				
Appropriate number of well care visits during foster care	Adolescents who received all recommended annual well-care visits during foster care	All adolescents	Recommendations for Preventive Pediatric Health Care [24]	For every portion of a year that a child spent in foster care, whether that time was continuous or not, at least one well care visit should have occurred
Appropriate adolescent immunizations	Adolescents who received at least one TdaP vaccination on or after their 10th birthday but before their 19th birthday and at least one Meningococcal vaccination on or after their 11th birthday but before their 19th birthday, whether received while in foster care or not	All adolescents	CDC 2013 Immunization Schedules [32]	TdaP or Td vaccinations both counted towards this measure
			HEDIS Adolescent Immunization Measure [29]	
Three Human Papilloma Virus (HPV) vaccinations in females	Three Human Papilloma Virus (HPV) vaccinations on or after the 9th birthday but before the 13th birthday, whether received while in foster care or not	Females only	HEDIS Human Papillomavirus Vaccine for Female Adolescents Measure [29]	
Appropriate number of BMI measurements	Adolescents who received all recommended annual BMI measurements during foster care	All adolescents	Recommendations for Preventive Pediatric Health Care [24]	For every portion of a year that a child spent in foster care, whether that time was continuous or not, at least one BMI measurement should have occurred
Appropriate number of drug use assessments	Adolescents who received all recommended annual drug use assessments during foster care	All adolescents	Recommendations for Preventive Pediatric Health Care [24]	For every portion of a year that a child spent in foster care, whether that time was continuous or not, at least one drug use assessment should have occurred
Appropriate number of alcohol use assessments	Adolescents who received all recommended annual alcohol use assessments during foster care	All adolescents	Recommendations for Preventive Pediatric Health Care [24]	For every portion of a year that a child spent in foster care, whether that time was continuous or not, at least one alcohol use assessment should have occurred
Appropriate number of sexually transmitted infection screenings	Adolescents who received all recommended annual chlamydia and gonorrhea screenings during foster care	All adolescents	Recommendations for Preventive Pediatric Health Care [24]	For every portion of a year that a child spent in foster care, whether that time was continuous or not, at least one chlamydia and at least one gonorrhea screening should have occurred
Appropriate number of depression screenings	Adolescents who received all recommended annual depression screenings during foster care	All adolescents	Recommendations for Preventive Pediatric Health Care [24]	For every portion of a year that a child spent in foster care, whether that time was continuous or not, at least one depression screening should have occurred

Chart reviews were performed by two staff members familiar with EHR data abstraction (Epic Systems Corporation, Wisconsin). An instruction document outlining the data elements and their common locations was created and used by both reviewers. Inter-rater reliability analyses were performed on the first 41 reviewed records of children within each age group to ensure reproducibility between data abstractors with excellent reproducibility [25, 26]. Study data were managed using REDCap data tool [27]. This study was approved by the NCH Institutional Review Board.

Because exact entry and exit dates for out of home care were often missing from EHRs, entry and exit dates were calculated in three different ways depending on the availability of data: 1) exact entry or exit dates were recorded whenever available, 2) the midpoint of an available date range (dates between health care visits wherein a child was documented to have entered/exited foster care) was used for entry or exit dates, and finally 3) the first well-care visit (or other documented healthcare encounter for adolescents) after entry was considered the entry date and the last well-care visit (or other documented healthcare encounter for adolescents) was considered the exit date when an exact date or date range was unavailable.

SACWIS is a “comprehensive automated case management tool that supports foster care and adoptions assistance case management practice.” [28] This system is intended to hold the official case record of all children currently or previously in out-of-home care in a state. Not all states use SACWIS, and there is substantial heterogeneity across states in the contents of SACWIS [28]. This study used data available in the Franklin County, Ohio SACWIS system in order to reflect the same population studied in the EHR review. A data extract from SACWIS was sent to the investigators containing the records of all children who were in custody during the study period and met inclusion criteria. Inclusion criteria were identical to those used for the EHR review, with the one exception that documentation of a well-care visit was not required. To determine whether data from the EHR and SACWIS could be reliably combined for analyses, we performed matching, using social security numbers when these were available in both the EHR and in SACWIS. As social security numbers were unavailable in one or both databases for approximately 70% of patients, when this number was unavailable we also considered records to be from the same child if they matched on all four of the following criteria: last name, first name, date of birth, and gender. A last name in the EHR was considered to match a last name in SACWIS if the first four characters were identical. Both the primary name and alias listed in SACWIS were considered. A first name in the EHR was considered to match a first name in SACWIS if the first four characters, either with or without symbols, were identical. Again, both the primary name and alias listed in SACWIS were considered.

Continuous variables were described with medians and interquartile ranges (IQR) as none were normally distributed. Categorical variables were described using frequencies and percentages. Quality measures were calculated in either the entire young child or the entire adolescent study cohort. We chose to review a sample of 400 EHRs for both the young child and adolescent EHR studies. The medical record numbers of all included children were sorted randomly such that the children whose charts were reviewed were a random sample of all children who could have been included.

In order to provide the most flexible information on the feasibility of obtaining the proposed quality measures from our data sources, two methods were used to calculate the proposed quality measures: proportions and observed-to-expected ratios. Most quality measures of pediatric healthcare focus on the former. Unfortunately, calculations of such often require steady denominators with fixed lengths of follow-up such as the number of children screened over the number of children eligible who were tracked for a full year. Because foster children cycle in and out of care, the denominator calculations may be ineffective in describing this unstable population.

Several quality measures were calculated as observed (received) to expected (recommended) ratios (O/E ratios) to better describe the actual quantity of recommended health care that was received by individual children, whereas the proportion measures indicate the percentage of children in the study cohort that received all recommended care. Weighted mean O/E ratios were calculated wherein each child’s individual O/E ratio was weighted by his or her total time spent in foster care during the study period. This weighting was performed because it enabled children who spent more time in foster care to contribute more to the calculated O/E measures. We used the AAP *Recommendations for Preventive Pediatric Health Care* as the guideline for determining the expected, or recommended, number of well-care visits and developmental screenings in the young children and the recommended number of well-care visits, BMI measurements, drug use assessments, alcohol use assessments, sexually transmitted infection screenings, and depression screenings in the adolescents [24]. For each measure, an individual O/E ratio was calculated for each child and then a weighted average of these individual O/E ratios was calculated to provide an appropriate average O/E for the entire cohort.

Continuous variables were described with medians and interquartile ranges (IQR). Categorical variables were described using frequencies and percentages. We also attempted to extract data on the same types of health care encounters that were examined in the chart reviews. SAS version 9.3 (SAS Institute Inc., NC) was used to analyze all data.

## Results

### Study of EHR data of children age 0–3 years

A total of 400 charts were reviewed. Twenty-five children were excluded from analyses because they did not meet inclusion criteria; 8 were without any well-care visits and 17 entered foster care prior to January 1, 2007. This left 375 patients to be included in analyses (Table 2). Overall, the median duration of time spent in foster care during the study period of 9.2 months (IQR 3.0–17.9) (Table 2). Around 76% of children had exact dates of entry but only 44.8% of children had both exact entry and exact exit dates recorded in their EHR. A quarter of the study population lacked documentation in their EHR of the reason for their first entry into foster care.

**Table 2:** Demographic, Entry, and Exit Related Characteristics of 0–3 Year Olds

	EHR	SACWIS
	*N* = 375	*N* = 1887
Age in months at first entry into foster care	6 (0, 16)	6 (0, 19)
Number of months in foster care from birth through age 3 years or Feb 28, 2013^a^	9.2 (3.0, 17.9)	8.4 (1.9, 17.0)
Exact dates for all entries into and exits from foster care	168 (44.8)	1887 (100)
Exact dates of all entries into foster care during study period	287 (76.5)	1887 (100)
Male	193 (51.5)	984 (52.2)
Race/Ethnicity		
White	129 (34.4)	713 (37.8)
Black/African-American	152 (40.5)	772 (40.9)
≥ 2 Races	39 (10.4)	223 (11.8)
Other	30 (8.0)	4 (2.1)
Unknown/Not documented	25 (6.7)	39 (2.1)
Hispanic	13 (3.5)	136 (7.2)
Reason for first entry into foster care^b^		
Neglect^c^	N/A	550 (29.2)
Dependency^d^	N/A	368 (19.5)
Parental drug or alcohol abuse	122 (32.5)	358 (19.0)
Physical abuse	37 (9.9)	311 (16.5)
Caretaker’s inability to cope	N/A	99 (5.3)
Incarceration of parents	15 (4.0)	49 (2.6)
Other	138 (36.8)	152 (8.1)
Not documented	96 (25.6)	0 (0)
Number of entries into foster care		
1	353 (94.1)	1734 (91.9)
2	22 (5.9)	138 (7.3)
3	0 (0)	15 (0.8)
Foster home changes during all foster care episodes		
None	322 (85.9)	1091 (57.8)
1	46 (12.3)	490 (26.0)
2	4 (1.1)	210 (11.1)
> 2	3 (0.8)	96 (5.1)
Still in foster care as of 3rd birthday or Feb 28, 2013		
Yes	114 (30.4)	219(11.61)
No, reunited	76 (20.3)	813 (43.1)
No, entered kinship care	63 (16.8)	336 (17.8)
No, adopted	44 (11.7)	314 (16.6)
Other	N/A	193 (10.2)
Unknown/Not documented	78 (20.8)	12 (0.6)

Data are shown as median (interquartile range) or frequency (%). ^a^Unknown dates of entry and exit in the EHR study cohort were estimated as described in the methods. For the calculation of months spent in foster care, a 30 day period was treated as a month. ^b^Some patients had multiple reasons for entry into foster care, all of which were extracted. However, only the primary reason for removal is available in a structured field in SACWIS. ^c^Neglect was not captured as a reason for entry into foster care in the EHR study. Rather, particular types of neglect or reasons for neglect were captured such as parental drug and alcohol abuse, abandonment, and parental developmental disability or mental illness. ^d^Dependency removals were for reasons not related to abuse or neglect, or in cases when parents could not care for their children but were not neglecting or abusing them (e.g. homelessness or death of parents in a car accident). This type of reason for entry was also not captured as a distinct category in the EHR study, but rather is incorporated within the “Other category”

Table 3 illustrates the performance of the proposed health care quality measures for young children within our EHR. Among 341 children in the study cohort with an exact date or date range of initial entry into foster care, we observed that 78.6% received a well-care visit within 30 days of entry. Over 79% of all children included in this study received the appropriate number of lead screenings, and 100% with traumatic brain injury (TBI) had a developmental screening within 3 months of their diagnosis. More than half of all children in foster care received the appropriate number of well-care visits (59.2%) and developmental screenings (57.9%) during foster care, and 83% received all of the recommended diphtheria, tetanus, pertussis vaccine (TDap), inactivated poliomyelitis (IPV), Haemophilus Influenzae Type b (HiB), and hepatitis b (HepB) vaccinations by age 1. Over 70% of children received the appropriate number of recommended vaccinations by age 2. Only about 1 in 5 children suspected of physical abuse received a follow-up skeletal survey after one was initially performed, and only 3.2% of transitions from foster home to foster home showed any evidence of a care coordination letter. Data from children who had exact dates of entry and exit revealed similar results for all quality measures (data not shown).

**Table 3:** Quality Measures in all Children Aged 0–3 Years

Proportion Measures			
	Numerator	Denominator	%
1st well-care visit within 30 days of initial entry into foster care^a^	268	341	78.6
Appropriate number of well care visits for their age during foster care^b^	190	321	59.2
Appropriate number of vaccinations by age 1 year^c^	112	135	83.0
Appropriate number of vaccinations by age 2 years^c^	82	114	71.9
Appropriate number of lead screenings for their age during foster care	153	193	79.3
Appropriate number of developmental screenings for their age during foster care^b^	186	321	57.9
Developmental screening during foster care within 3 months after documentation of TBI	4	4	100.0
Follow up skeletal survey after receiving an initial skeletal survey	7	33	21.2
Care coordination letters at foster home changes	2	63	3.2
Observed to Expected Ratio Measures			
	# of children contributing to the weighted average^d^	Weighted Average Observed/Expected Ratio	
Appropriate number of well care visits for their age during foster care^b^	321	0.90	
Appropriate number of developmental screenings for their age during foster care^b^	321	0.94	

^a^Only children who had an exact date of entry or a date range for entry were included in this measure

^b^54 children who had zero expected well-care visits for their age while in foster care (per AAP Bright Futures Guidelines) are not included in these measures

^c^Vaccinations included DTaP, IPV, HiB, and HepB for age 1 and DTaP, IPV, HiB, HepB, MMR, and VZV vaccinations for age 2

^d^The denominator of the O/E measures indicates how many individuals’ O/E ratios were included in the calculation of the overall weighted average O/E ratio

O/E ratios were calculated for the well-child visit and developmental screening measures (Table 3). On average, children received 90% of their recommended well care visits while in foster care and 94% of their recommended developmental screenings while in foster care. These O/E measures, for the reasons already discussed, are higher than their analogous proportion measures.

### SACWIS data of children age 0–3 years

A total of 1887 children age 0–3 years with records in SACWIS met our inclusion criteria. (Table 2) Demographic, entry and exit characteristics were similar between the EHR and SACWIS study cohorts, but one key difference between data sources is the consistent documentation of entry and exit dates in SACWIS (100% in SACWIS vs. 44.8% in the EHRs). In addition, SACWIS contains a greater amount of detail regarding foster care history compared to data from the EHR. Unfortunately, SACWIS contains far less detail on the health care provided to children in foster care than EHRs. After reviewing the SACWIS records of a matched sample of the EHR study cohort, it was found that SACWIS was not a viable resource for medical data (data not shown).In addition, only approximately 50% (198/375) of patients with EHR data could be matched across the two data sources.

### EHR data of adolescents

A total of 401 charts were reviewed. Two were excluded because they did not have any well-care visits while in foster care during the study period. This left 399 patients. Table 4 depicts the demographic and entry and exit related characteristics of both the EHR and SACWIS study populations. Overall, the median duration of time spent in foster care during the study period was 10.1 months (IQR 2.5–21.0) (Table 4). Almost 75% of children had exact dates of entry but only 21.8% of children had both exact entry and exact exit dates recorded in their EHR. The most frequently cited reason for first entry into foster care was a child’s behavior problem (30.3%). However, almost 40% lacked documentation in their EHR of the reason for their first entry into foster care.

**Table 4:** Demographic, Entry, and Exit Related Characteristics of Adolescents Aged 12–18 Years

	EHR	SACWIS
	*N* = 399	*N* = 3674
Age in years at entry into the first period in foster care that overlapped with or occurred entirely during the study period	15 (13, 16)	15 (14, 16)
Number of months in foster care while aged 12–18 years during the study period^a^	10.1 (2.5, 21.0)	7.0 (2.3, 14.0)
Exact dates for all entries into and exits from foster care	87 (21.8)	3674 (100)
Exact dates of all entries into foster care during study period	299 (74.9)	3674 (100)
Male	205 (51.4)	2097 (51.7)
Race/Ethnicity		
White	119 (29.8)	1272 (34.6)
Black/African-American	240 (60.2)	1952 (52.4)
≥ 2 Races	18 (4.5)	288 (7.8)
Other	17 (4.3)	223 (11.8)
Unknown/Not documented	5 (1.3)	22 (0.6)
Hispanic	20 (5.0)	152 (4.1)
Primary reason for entry into the first period in foster care that overlapped with or occurred entirely during the study period^b^		
Child Behavior Problem	121 (30.3)	1073 (29.2)
Delinquency^c^	N/A	1056 (28.7)
Dependency^d^	N/A	275 (7.5)
Neglect	23 (5.8)	272 (7.4)
Physical abuse	28 (7.0)	205 (5.6)
Sexual abuse	29 (7.3)	82 (2.2)
Other	45 (11.3)	711 (19.4)
Not documented	153 (38.3)	0 (0)
Number of distinct episodes in foster care during study period		
1	369 (92.5)	2890 (78.7)
2	26 (6.5)	590 (16.1)
3	4 (1.0)	152 (4.1)
> 3	0 (0)	42 (1.1)
Foster home changes during all foster care episodes^e^		
None	258 (64.7)	1554 (42.3)
1–2	118 (29.6)	1307 (35.6)
3–4	18 (4.5)	437 (11.9)
> 4	5 (1.3)	376 (10.2)
Still in foster care as of 19th birthday or Oct 31, 2013 (whichever came first)		
Yes	66 (16.5)	163 (4.4)
No, reunited	49 (12.3)	2033 (55.3)
No, entered kinship care	13 (3.3)	353 (9.6)
No, adopted	10 (2.5)	41 (1.1)
No, aged out, living independently, or emancipated	13 (3.3)	650 (17.7)
No, runaway	2 (0.5)	243 (6.6)
Other	0 (0)	191 (5.2)
Unknown/Not documented	246 (61.7)	12 (0.6)

Data are shown as median (interquartile range) or frequency (%). ^a^Unknown dates of entry and exit in the EHR study cohort were estimated as described in the methods. For the calculation of months spent in foster care, a 30 day period was treated as a month. ^b^Only the primary reason for removal was recorded in both the EHR and SACWIS data. ^c^Delinquency was not captured as a reason for entry into foster care in the EHR study. ^d^Dependency removals were for reasons not related to abuse or neglect, or in cases when parents could not care for their children but were not neglecting or abusing them (e.g. homelessness or death of parents in a car accident). This type of reason for entry was also not captured as a distinct category in the EHR study, but rather is incorporated within the “Other category”. ^e^Only transitions into or out of standard foster homes or group homes were included. Transitions into and out of other placement settings were not included

Table 5 illustrates the performance of the proposed adolescent health care quality measures within our EHR. More than 3/4ths received the appropriate number of annual well-care visits and recommended TdaP and Meningococcal vaccinations. However, less than 1 in 10 girls had documentation in their EHR of having received a full 3-dose course of the human papillomavirus (HPV) vaccine. Over 90% of adolescents had documentation of an annual BMI. Over 75% had documentation of annual drug use assessments, but only 33.8% had documentation of annual alcohol use assessments. Less than half of adolescents were screened annually for both chlamydia and gonorrhea, and less than 25% were screened annually for depression.

**Table 5:** Quality Measures in Adolescents Aged 12–18 Years

Proportion Measures			
	Numerator	Denominator	%
Appropriate number of well care visits for their age during foster care	329	399	82.5
Appropriate adolescent immunizations^a^	311	399	77.9
Three HPV vaccinations in females	26	194	13.4
Appropriate number of BMI measurements	368	399	92.2
Appropriate number of drug use assessments	300	399	75.2
Appropriate number of alcohol use assessments	135	399	33.8
Appropriate number of sexually transmitted infection screenings	161	399	40.4
Appropriate number of depression screenings	94	399	23.6
Observed to Expected Ratio Measures			
	# of children contributing to the weighted average^b^	Weighted Average Observed/Expected Ratio	
Appropriate number of well care visits for their age during foster care	399	0.96	
Appropriate number of BMI measurements	399	1.82	
Appropriate number of drug use assessments	399	0.89	
Appropriate number of alcohol use assessments	399	0.40	
Appropriate number of sexually transmitted infection screenings	399	0.67	
Appropriate number of depression screenings	399	0.28	

^a^Vaccinations included TdaP and meningococcal vaccinations. ^b^The denominator of the O/E measures indicates how many individuals’ O/E ratios were included in the calculation of the overall weighted average O/E ratio

O/E ratios were calculated for all of the same events for which proportion measures were calculated, with the exception of the immunization measures (Table 5). On average, adolescents received 96% of their recommended well care visits while in foster care. On average, they received 89% of the recommended number of drug use assessments for the time they spent in foster care, but the rates of screening for alcohol use, sexually transmitted infections, and depression were considerably lower.

### SACWIS data of adolescents

A total of 3674 adolescents aged 12–18 years with records in SACWIS met our inclusion criteria (Table 4). Demographic, entry and exit characteristics were similar between the EHR and SACWIS study cohorts, though the proportion of adolescents with undocumented reasons for entry into and exit from foster care in their EHR makes it challenging to compare these characteristics between cohorts. SACWIS contains minimal detail on the health care provided to children in foster care when compared to EHRs.

## Discussion

Documentation of data important to the tracking and optimization of the health care of children and adolescents in foster care is frequently incomplete and difficult to find in either EHRs or SACWIS in our patient population. However, despite their limitations, EHRs and SACWIS can be useful data sources for the calculation of some important measures of quality of care in the foster care population, and would be even more useful if certain important data elements were more consistently available and easily extractable from each database. Alternatively, individual level matching across platforms may allow for the optimal methods by which to assess these measures.

Manual review to calculate our proposed foster care quality measures was laborious. The majority of information was located in free text fields and scanned documents rather than structured fields. Many important data elements, specifically the reasons for initial entry into foster case and the entry and exit dates from foster care, were often missing, and this lack of documentation proved to be limiting factors in data abstraction. The accuracy of nearly all of our proposed health care quality measures is contingent upon this critical information. Similar issues were identified for SACWIS data. While demographics and entry and exit characteristics were found in discrete fields, all other information of interest to this study was located in free text fields. This required study investigators to visually examine text notes. Even after this task was performed, it was found that the quantity of information and detail on medical care in SACWIS was far less than from the EHRs.

Identifying the best method to calculate the proposed quality measures revealed the complexity of EHR data abstraction and quality measure development for children in foster care. For example, the Healthcare Effectiveness Data and Information Set (HEDIS) Well-Child Visit measure at 15 months of age would have minimal utility in the foster care population as it requires continuous enrollment for 12 months prior to age 15 months as an inclusion criteria [29]. The O/E ratios examined in this study seemed to be a viable option for the calculation of quality of care measures in the foster care population, primarily because every child can be included in the calculation of these measures regardless of their length of stay or number of episodes in foster care. In addition, children who spend more time in foster care appropriately contribute more to these measures than children who spend less time in foster care. The results in Tables 3 and 5 indicate that select quality measures appear better when calculated using O/E ratios rather than proportions, namely because all health care events contribute to the O/E measures whereas with the proportion measures, a child is counted in the numerator only if the ideal number of events of interest occurred. Admittedly however, the greater mathematical complexity of the weighted average O/E measures, compared to simple proportions, may limit their widespread use.

Considering the challenges we encountered in this study, modification of current EHRs, the use of another data source, or combination of data sources may improve the feasibility of foster care quality measures. An ideal EHR format specific to children was recently proposed [30]. The format provides specific elements and requirements that could be added to current EHRs to enhance the care of children, especially those enrolled in Medicaid and in the care of child welfare [30]. These recommendations include system capacity to store and display 1) whether the child has ever been in out-of home care 2) information about the dates of the out-of-home care and 3) information on the child’s history of abuse or neglect. In addition, the SACWIS data system used by child welfare agencies could also be useful. Although its current use varies by state, it is intended to be a comprehensive database that supports the efforts of case workers to assist children in out of home care [28]. The availability of exact dates of entry into foster care and exit from foster care in SACWIS and the availability of accurate data on health care received in the EHR could, together in a combined database, enable the calculation of more accurate health care quality measures than those presented here. However, a higher match rate than we found would be necessary to make such a combined database useful.

## Conclusion

Extraction of data to test foster care quality measures is not currently feasible in a single institution EHR, even though we conducted this study at a large, free-standing children’s hospital with a longstanding commitment to electronic health records, nor is it feasible in a metropolitan county’s SACWIS data. Most proposed quality measures tested did not achieve high adherence as recommended by current guidelines, but it is difficult to tell to what extent missing data elements such as entry and exit dates contributed to these results. Because the quality of information is important to improve patient care, testing foster care quality measures in SACWIS or an augmented EHR that utilizes the children’s EHR format may be a better alternative, and subsequently may yield more reliable results [31].

## Abbreviations

BMI: Body mass index
CHIPRA: Children’s Health Insurance Program Reauthorization Act
EHR: Electronic health record
HEDIS: Healthcare effectiveness data and information set
HepB: Hepatitis b vaccine
HiB: Haemophilus Influenzae Type b vaccine
HPV: Human papillomavirus
IPV: Inactivated Poliomyelitis vaccine
IQR: Interquartile ranges
NCH: Nationwide Children’s Hospital
NCINQ: National Collaborative for Innovation in Quality Measurement
O/E ratios: Observed to expected ratios
PCP: Primary care physician
PQMP: Pediatric Quality Measures Program
SACWIS: Statewide Automated Child Welfare Information System
TBI: Traumatic brain injury
TdaP: Tetanus,Diphtheria, Pertussis vaccine
